# Computational Identification of Blood–Brain Barrier-Permeant Microbiome Metabolites with Binding Affinity to Neurotransmitter Receptors in Neurodevelopmental Disorders

**DOI:** 10.3390/molecules31020366

**Published:** 2026-01-20

**Authors:** Ricardo E. Buendia-Corona, María Fernanda Velasco Dey, Lisset Valencia Robles, Hannia Josselín Hernández-Biviano, Cristina Hermosillo-Abundis, Lucila Isabel Castro-Pastrana

**Affiliations:** 1 Departamento de Ciencias Químico-Biológicas, Universidad de las Américas Puebla, Puebla 72810, Mexico; ricardo.buendiaca@udlap.mx; 2 Facultad de Psicología, Benemérita Universidad Autónoma de Puebla, Puebla 72500, Mexico; vd202474904@alm.buap.mx (M.F.V.D.); vr202462433@alm.buap.mx (L.V.R.); ana.hermosilloa@correo.buap.mx (C.H.-A.); 3 Facultad de Ciencias Biológicas, Benemérita Universidad Autónoma de Puebla, Puebla 72522, Mexico; hannia.hernandezb@alumno.buap.mx; 4 Escuela de Ciencias, Universidad de las Américas Puebla, Ex Hacienda Sta. Catarina Mártir S/N, San Andrés Cholula 72810, Mexico

**Keywords:** microbiota, blood–brain barrier, computer neural network, molecular docking simulation, neurodevelopmental disorders

## Abstract

The gut microbiome produces thousands of metabolites with potential to modulate central nervous system function through peripheral or direct neural mechanisms. Tourette syndrome, attention-deficit/hyperactivity disorder, and autism spectrum disorder exhibit shared neurotransmitter dysregulation and microbiome alterations, yet mechanistic links between microbial metabolites and receptor-mediated neuromodulation remain unclear. We screened 27,642 microbiome SMILES metabolites for blood–brain barrier permeability using rule-based SwissADME classification and a PyTorch 2.0 neural network trained on 7807 experimental compounds (test accuracy 86.2%, AUC 0.912). SwissADME identified 1696 BBB-crossing metabolites following Lipinski’s criteria, while PyTorch classified 2484 metabolites with expanded physicochemical diversity. Following 3D conformational optimization (from SMILES) and curation based on ≤32 rotatable bonds, molecular docking was performed against five neurotransmitter receptors representing ionotropic (GABRA2, GRIA2, GRIN2B) and metabotropic (DRD4, HTR1A) receptor classes. The top 50 ligands across five receptors demonstrated method-specific BBB classification (44% SwissADME-only, 44% PyTorch-only, 12% overlap), validating complementary prediction approaches. Fungal metabolites from *Ascomycota* dominated high-affinity top ligands (66%) and menaquinone MK-7 showed broad phylogenetic conservation (71.4% of phylum). Our results establish detailed receptor–metabolite interaction maps, with fungal metabolites dominating high-affinity ligands, challenging the prevailing bacterial focus of the microbiome and providing a foundation for precision medicine and a framework for developing microbiome-targeted therapeutics to address clinical needs in neurodevelopmental disorders.

## 1. Introduction

The microbiota–gut–brain axis represents a bidirectional communication network whereby intestinal microbiota influences central nervous system function through multiple pathways [1,2]. Critically, microbial metabolites can exert neuromodulatory effects through two distinct routes: peripheral mechanisms involving gut–brain axis signaling through vagal afferents, enteroendocrine cells, and immune mediators, or direct central mechanisms requiring blood–brain barrier traversal to interact with neural tissue [2,3]. Microbial metabolites include short-chain fatty acids, neurotransmitter precursors, tryptophan derivatives, and bile acid metabolites, each with distinct physicochemical properties determining their site of action [4].

Neurodevelopmental disorders (NDD) represent a group of conditions characterized by impaired brain and nervous system development, manifesting in childhood with lifelong persistence. Tourette syndrome (TS), attention-deficit/hyperactivity disorder (ADHD), and autism spectrum disorder (ASD) share remarkable clinical, genetic, and neurobiological overlap [5,6]. TS is characterized by repetitive involuntary movements and vocalizations, with approximately 50–60% of patients exhibiting comorbid ADHD and 20–30% showing ASD features [7]. ADHD affects 5–7% of children worldwide, presenting with persistent inattention, hyperactivity, and impulsivity, with autism co-occurring in 30–50% of cases. ASD, with prevalence approaching 1 in 36 children, encompasses social communication deficits and restricted repetitive behaviors, frequently accompanied by attention difficulties [8]. This substantial clinical overlap, combined with shared genetic susceptibility loci and familial aggregation patterns, suggests convergent underlying pathophysiological mechanisms rather than distinct disease entities [5,9]. The heterogeneity within each disorder and variability in treatment response further suggest that patient subgroups may harbor distinct neurobiological subtypes requiring personalized therapeutic approaches.

At the neurobiological level, TS, ADHD, and ASD converge on dysregulation of key neurotransmitter systems operating through both ionotropic and metabotropic receptor classes. Ionotropic receptors—ligand-gated ion channels including GABA, glutamate AMPA, and glutamate NMDA receptors—mediate rapid synaptic transmission through direct ion flux, with millisecond-scale kinetics governing excitatory and inhibitory neurotransmission balance. Metabotropic receptors—G-protein coupled receptors including dopamine and serotonin receptor subtypes—produce slower, more sustained modulatory effects through second messenger cascades affecting neuronal excitability, synaptic plasticity, and gene expression [10,11,12,13]. This dual receptor architecture provides multiple regulatory nodes where microbiome metabolites could exert neuromodulatory effects, with consequences depending on receptor type, brain region, developmental stage, and baseline activity state [14].

Dopaminergic dysfunction, operating primarily through metabotropic D1-like and D2-like receptor families, is central to all three conditions. TS involves cortico-striato-thalamo-cortical circuit hyperactivity, with excessive dopamine release in striatal regions contributing to tic generation through D2 receptor-mediated disinhibition of motor programs [15,16]. ADHD shows reduced dopamine signaling in the prefrontal cortex and striatum, affecting attention, motivation, and executive function, with genetic variants in dopamine D4 (DRD4) and D5 receptors conferring risk. ASD exhibits altered dopamine receptor expression in reward pathways and social cognition circuits, with dopaminergic dysfunction contributing to restricted interests and social motivation deficits. GABAergic abnormalities, operating through ionotropic GABAA receptors, affect excitatory/inhibitory balance across all three disorders. The excitatory/inhibitory imbalance represents a hallmark of ASD pathophysiology, with postmortem studies revealing reduced GABAergic interneuron density and altered GABA receptor subunit expression. GABAergic dysregulation in TS contributes to motor disinhibition, while ADHD involves prefrontal GABAergic deficits affecting cognitive control. Serotonergic alterations, operating through multiple metabotropic receptor subtypes (5-HT1A, 5-HT2A, 5-HT2C), contribute to behavioral symptoms across conditions, affecting impulsivity and emotional regulation in ADHD, repetitive behaviors and compulsivity in TS and ASD, and social cognition and sensory processing in ASD [12,17]. Glutamatergic system dysfunction, operating through both ionotropic (AMPA, NMDA) and metabotropic glutamate receptors, underlies synaptic plasticity deficits in ASD and cognitive impairments in ADHD, with genetic studies identifying risk variants in glutamate receptor genes. The involvement of both ionotropic and metabotropic receptor classes across multiple neurotransmitter systems provides a mechanistic framework for understanding how microbiome metabolites with diverse binding affinity to these receptors could modulate neurotransmission in NDDs.

Recent research has documented significant alterations in gut microbiome composition in patients with TS, ADHD, and ASD compared to neurotypical controls [18]. Children with ASD show reduced microbial diversity, altered *Firmicutes*/*Bacteroidetes* ratios, and distinct metabolomic signatures in fecal samples and blood plasma. Metabolomic analyses reveal elevated levels of phenolic compounds, altered tryptophan metabolites, modified short-chain fatty acid profiles, and abnormal amino acid metabolism. ADHD patients exhibit dysbiosis characterized by decreased abundance of certain *Bacteroides* and *Prevotella* species and altered short-chain fatty acid production [19]. Emerging TS cohort data reveal microbiome differences correlating with tic severity [11]. Several studies have detected abnormal concentrations of microbiome-derived metabolites in biological fluids of NDD patients, suggesting microbial dysbiosis contributes to pathophysiology through metabolic mechanisms [20,21]. Animal studies provide mechanistic support: germ-free mice exhibit behavioral abnormalities and neurotransmitter imbalances reversible through microbiome reconstitution, while fecal microbiota transplantation from ASD patients transfers behavioral phenotypes to recipient mice, establishing causal relationships between microbiome composition and neurodevelopmental outcomes [22,23,24].

Despite growing recognition of microbiome alterations in neurodevelopmental disorders, the specific molecular mechanisms through which microbial metabolites influence neurotransmitter receptor function remain poorly characterized. Systematic assessment of which metabolites possess BBB permeability and binding affinity to disorder-relevant receptors has not been comprehensively performed [25,26,27]. Understanding which metabolites operate through which pathways is essential for rational therapeutic development. This knowledge gap represents a critical barrier to developing rational microbiome-targeted therapeutic interventions for neurodevelopmental disorders. Identifying BBB-crossing metabolites with specific receptor binding profiles would enable selection of probiotic strains producing metabolites targeting particular therapeutic needs. For instance, patients with predominantly dopaminergic dysfunction (attention deficits in ADHD) might benefit from strains producing metabolites with DRD4 binding affinity, while patients with excitatory/inhibitory imbalance (characteristic of ASD) might benefit from strains producing GABAergic or glutamatergic modulators [28,29]. Metabolites exhibiting promiscuous binding across multiple receptor types offer therapeutic value, potentially addressing diverse symptom domains simultaneously through multi-receptor modulation. Such metabolites could be especially beneficial given the substantial comorbidity and symptom overlap across TS, ADHD, and ASD. Furthermore, metabolomic profiling of individual patients could enable precision medicine approaches where probiotic combinations are personalized based on each patient’s specific metabolite deficiencies or excesses and neurotransmitter dysfunction [30,31]. This represents a fundamentally different paradigm from current one-size-fits-all probiotic formulations, potentially improving efficacy through individualized targeting. The present study aimed to address this knowledge gap through computational screening, stratifying microbiome metabolites by site of action and receptor binding affinity. We systematically predicted BBB-permeant candidates from the microbiome metabolite database MiMeDB using complementary BBB permeability-classifying approaches. To assess potential neuromodulatory activity, we evaluated binding affinity of BBB-permeant metabolites to a panel of neurotransmitter receptors using molecular docking. This panel included key receptors representing both ionotropic (GABRA2, GRIA2, GRIN2B) and metabotropic (DRD4, HTR1A) classes, selected for their fundamental and well-characterized roles in regulating synaptic excitation, inhibition, and neuromodulation. This selection provides a broad coverage of major neurotransmitter systems (GABAergic, glutamatergic, dopaminergic, and serotonergic) and allows for the exploration of potential metabolite interactions with distinct signaling modalities. While dysfunction in these specific receptors has been implicated in various brain disorders, including NDDs, which served as an initial motivator for this study, their primary relevance here is their established, central function in central nervous system (CNS) communication.

Our analysis identified metabolites with receptor-specific versus multitarget binding profiles, establishing a framework for rational probiotic selection based on targeted therapeutic needs. Collectively, these findings establish detailed receptor–metabolite interaction maps. This work provides a direct foundation for precision medicine approaches, where patient-specific metabolomic profiling could potentially inform personalized probiotic or dietary formulations, a future direction for addressing individual neurotransmitter dysfunction patterns.

## 2. Results

### 2.1. Comparison Between SwissADME and PyTorch Classification of BBB-Permeant Metabolites

**Figure 1:**
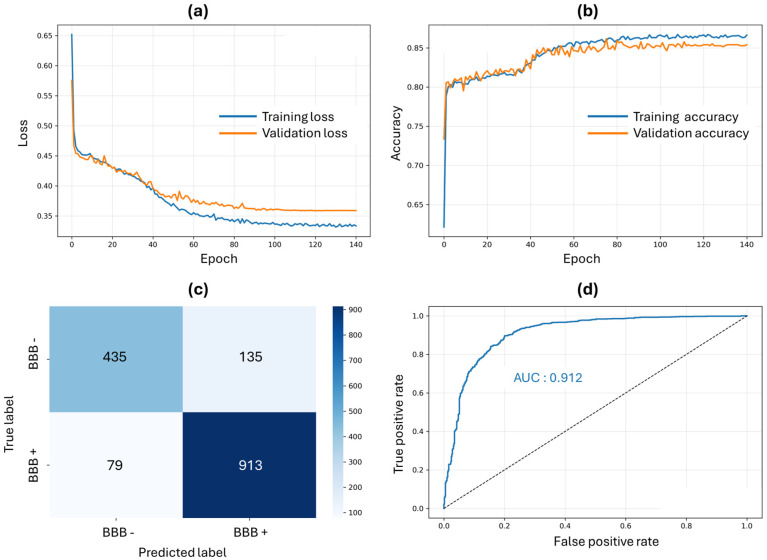
PyTorch neural network training dynamics and performance evaluation for BBB-crossing classification. (**a**) Training (blue) and validation (orange) loss curves over 140 epochs, showing rapid initial convergence and stabilization without overfitting. (**b**) Training and validation accuracy progression, achieving 0.87 and 0.85 accuracy, respectively. (**c**) Confusion matrix for test set predictions (*n* = 1562), demonstrating 913 true positives, 435 true negatives, 135 false positives, and 79 false negatives, corresponding to 86.2% overall accuracy. Gradient in blue indicates higher frequency of results. (**d**) Receiver operating characteristic (ROC) curve with an area under the curve (AUC) of 0.912, indicating high discriminatory performance. The dashed line represents a random classifier baseline.

Two classification methods were employed, as stated in the methodology section. The SwissADME web platform was used to predict BBB permeability. Compounds were classified as BBB-permeant when their SMILES notation placed them within the ‘yolk’ region of the BOILED-Egg model, based on log*p* and total polar surface area (TPSA) values. We also trained a PyTorch neural network for BBB classification using the experimental database BBB_DB (*n* = 7807 compounds) based on the nine features described in the Section 4. The resulting performance characteristics are presented in Figure 1.

Figure 1a shows the training and validation loss curves over 140 epochs, exhibiting rapid convergence during the initial training phase. Both training (blue) and validation (orange) losses decreased sharply within the first 20 epochs followed by gradual stabilization. Figure 1b presents the corresponding accuracy metrics throughout the training process. The model achieved rapid improvement in classification accuracy, with both training (blue) and validation (orange) reaching 0.87 final accuracy for the training, while validation accuracy stabilized at 0.85. The minimal divergence between training and validation accuracy curves suggests effective regularization and good model generalization. Figure 1c,d display the test classifier results and predictive performance with the confusion matrix and receiver operating characteristic (ROC) curve respectively. The model correctly classified 913 BBB-permeant metabolites (true positives) and 435 non-crossing metabolites (true negatives). This corresponds to an overall test accuracy of 86.2%, with higher sensitivity (92%) than specificity (76.3%), indicating the model’s preference for identifying BBB-permeant compounds. Discrimination capability is also represented with an area under the curve (AUC) of 0.912. To evaluate the relative contribution of each molecular descriptor to the PyTorch BBB classifier predictions, permutation-based importance analysis was performed according to the Section 4. Results in Figure S3 demonstrate that TPSA exhibited the highest importance score (~0.125), indicating that disruption of this feature most substantially impaired classification accuracy. Secondary predictive contributions were observed for HBDs (hydrogen bond donors), aromatic atoms, number of rings, log*p*, and molecular weight (importance scores ~0.02–0.03). Features including rotatable bonds, HBAs (hydrogen bond acceptors), and atom count showed minimal individual importance (<0.01). Verification of overlap between MiMeDB and BBB_DB datasets identified 33 molecules present in both databases, representing only 0.11% of the complete MiMeDB collection (*n* = 27642, accessed September 2025). During our dataset splitting procedure, these overlapping compounds were randomly distributed across training (*n* = 23), validation (*n* = 2), and test sets (*n* = 8). From these distributions, 10 overlapping compounds were held out from model training, either in the test set (used only for final evaluation) or validation set (used only for hyperparameter tuning). Furthermore, the 23 overlapping compounds in the training set represent only 0.08% of the total MiMeDB and 0.42% of the total training set (*n* = 5645), a proportion too small to significantly bias model performance or compromise the model’s generalization to the broader microbiome metabolite chemical space.

**Figure 2:**
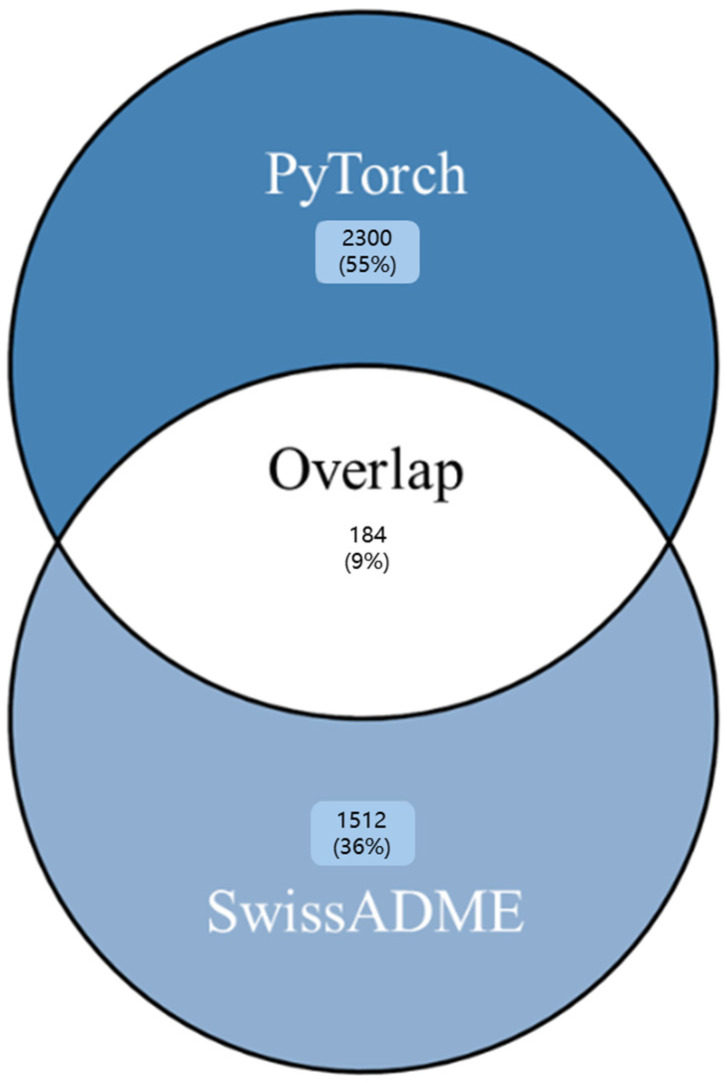
Venn diagram showing the overlap in blood–brain barrier (BBB)-permeant classification between the SwissADME model and the PyTorch neural network. Percentages represent the proportion of predicted BBB-permeant metabolites.

Both classification approaches were utilized to maximize chemical space coverage in subsequent molecular docking studies. From complete MiMeDB (*n* = 27,642, September 2025), the SwissADME classification yielded 1696 predicted BBB-permeant metabolites, while the PyTorch model identified 2484, with 184 metabolites classified by both methods (Figure 2). Rather than restricting analysis to this overlap, we proceeded with the union of both prediction sets (4180 unique metabolites) to capture metabolites that may traverse the BBB through either passive diffusion (captured by SwissADME) or alternative mechanisms such as carrier-mediated transport (captured by PyTorch).

**Figure 3:**
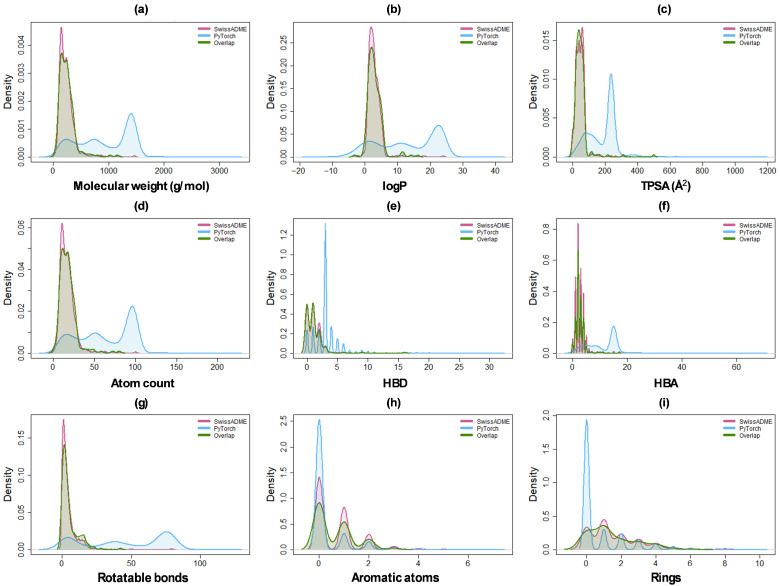
Comparative density distribution of key physicochemical descriptors for BBB-permeant metabolites. Density plots illustrate the distribution of nine molecular properties across three groups: SwissADME-predicted (pink), PyTorch-predicted (blue), and the high-confidence overlap (green). The descriptors shown are (**a**) molecular weight (g/mol), (**b**) log*p*, (**c**) total polar surface area (TPSA), (**d**) atom count, (**e**) hydrogen bond donors (HBD), (**f**) hydrogen bond acceptors (HBA), (**g**) rotatable bonds, (**h**) aromatic atoms, and (**i**) number of rings.

The distribution of molecular descriptors for BBB-crossing metabolites identified by SwissADME, PyTorch, and their overlap (metabolites classified as BBB-crossing by both methods) reveals distinct physicochemical profiles and method-specific biases (Figure 3 and Table 1). Figure 3a illustrates the molecular weight distributions, showing that SwissADME (pink) identified predominantly low-molecular-weight metabolites with a sharp peak around 200 g/mol (mean of 243.1 ± 146.5 g/mol), while PyTorch (blue) classified a broader distribution including a substantial population of higher-molecular-weight compounds (mean = 928.7 ± 478.0 g/mol). The overlap set (green) closely resembled the SwissADME distribution. Figure 3b presents the log*p* distributions. SwissADME showed a narrow distribution centered near neutral log*p* values (mean of 2.9 ± 2.2), indicating selection of moderately lipophilic metabolites. In contrast, PyTorch exhibited a broader distribution (mean = 12.9 ± 9.3) extending to log*p* values above 30, encompassing both hydrophilic and highly lipophilic metabolites. Overlap metabolites aligned closely with SwissADME. This pattern consistently repeats in total polar surface area (TPSA), atom count and rotatable bonds (Figure 3c,d,g). While both SwissADME and PyTorch rely on low hydrogen bond donors (HBD), high hydrogen bond acceptors (HBA) are allowed by PyTorch (mean of 11 ± 5.5). The number of rotatable bonds has a remarkable difference, with PyTorch (mean of 45 ± 30) able to allow very flexible molecules to cross the BBB. Both methods have a similar distribution of aromatic atoms and number of rings, resulting in an overlap in metabolite density.

Overall, these distributions reveal that SwissADME applies conservative physicochemical criteria. To associate this selection with traditional medicinal chemistry rules, Lipinski’s Rule of Five (Ro5) (molecular weight ≤ 500 g/mol, log*p* ≤ 5, HBD ≤ 5, HBA ≤ 10 [32]) was applied for both classifiers and is plotted in Figure S1, where SwissADME showed better Ro5 compliance, suggesting preference for traditional drug-like molecules.

**Figure 4:**
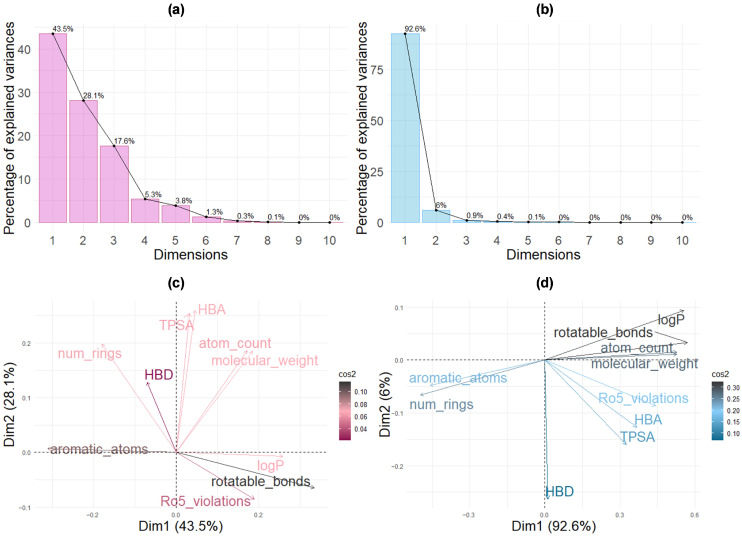
Principal component analysis comparing chemical descriptor distributions between SwissADME and PyTorch BBB-crossing classification models. (**a**,**b**) Scree plots showing variance explained by each PC dimension. (**c**,**d**) Biplots of PC1 vs. PC2 with descriptor loadings (arrows) and representation quality (cos^2^ color gradient).

To elucidate the interpretability of each classification approach, principal component analysis (PCA) was performed on the chemical descriptors using Spearman correlation matrices with scaled data normalization (Figure 4). SwissADME-classified metabolites exhibit multi-dimensional variance structure despite the model’s reliance on only two explicit parameters (log*p* and TPSA). Three principal components were required to capture 89.2% of total variance (PC1: 43.5%, PC2: 28.1%, PC3: 17.6%), with dispersed descriptor loadings and low representation quality (cos^2^: 0.04–0.10) (Figure 4a,c). This fragmented structure indicates that rule-based filtering produces chemically heterogeneous outputs without coherent organizational principles, effectively operating as a functional black box from the user’s perspective. In contrast, PyTorch-classified metabolites display a highly interpretable uni-dimensional variance structure, with PC1 alone accounting for 92.6% of total variance (Figure 4b). The tight clustering of chemical descriptors (Figure 4d) with superior representation quality (cos^2^: 0.10–0.30) demonstrates that the network learned an integrated “molecular complexity” factor that coherently combines size (molecular weight, atom count, number of rotatable bonds), lipophilicity (log*p*), polarity (TPSA, HBA, HBD), and aromaticity (number of rings and number of aromatic atoms). The PyTorch model thus identifies compounds spanning a broad spectrum of this complexity continuum, including larger, more lipophilic metabolites that achieve BBB penetration through mechanisms beyond simple passive diffusion. That is why the PyTorch approach identifies compounds spanning a broad spectrum of these complexity descriptors, including large, lipophilic metabolites that would be rejected by traditional filters but may nevertheless achieve BBB penetration through alternative mechanisms such as active transport.

### 2.2. Molecular Screening for 5 Neurotransmitter Receptors

**Figure 5:**
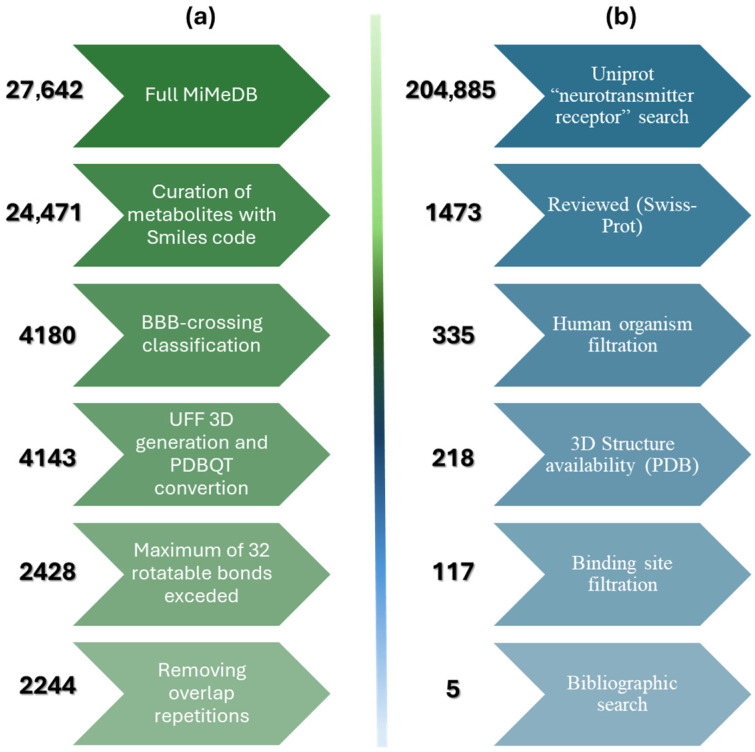
Filtering workflow for molecular screening preparation. (**a**) Ligand pipeline: MiMeDB metabolites (27,642) filtered through SMILES curation, BBB prediction including both PyTorch and SwissADME, 3D optimization, flexibility screening (≤32 rotatable bonds), and duplication, achieving 2244 final compounds. (**b**) Receptor pipeline: UniProt neurotransmitter receptors (204,885) filtered through database curation (Swiss-Prot), human organism restriction, PDB structure availability, binding site annotation, and disorder relevance, achieving 5 final targets.

The flow of data curation of ligands (BBB-crossing metabolites classified) and receptors is represented in Figure 5. During 3D optimization and PDBQT conversion, 37 metabolites failed due to invalid SMILES strings that could not be processed by OpenBabel 3.1.1 or contained chemical moieties incompatible with UFF parameterization; these were excluded from docking. A key technical consideration in our study involved the limitations of the molecular docking software used. AutoDock Vina 1.1.2 exhibits reduced accuracy when ligands contain more than 32 rotatable bonds [33]. To ensure the reliability of the resulting docking scores, we implemented a stringent curation step that excluded metabolites exceeding this 32-bond threshold. This procedure effectively refined the dataset for subsequent molecular screening, focusing only on compounds whose conformational sampling could be accurately modeled.

The selection of target receptors was based on their established roles in NDDs such as TS, ADHD, and ASD. Specifically, we targeted five key neurotransmitter receptors representing distinct ionotropic and metabotropic classes: the gamma-aminobutyric acid receptor subunit alpha 2 (GABRA2) [34,35,36,37], dopamine receptor 4 (DRD4) [38,39,40,41,42], serotonin receptor 1A (HTR1A) [43,44,45], alpha-amino-3-hydroxy-5-methyl-4-isoxazolepropionic acid, AMPA receptor 2 (GRIA2) [46,47], and the ionotropic glutamate receptor, NMDA 2B (GRIN2B) [48,49].

PDB IDs for each receptor are given in Table 2. All selected structures were experimentally determined by X-ray crystallography (5WUI and 3RN8) or cryo-electron microscopy (8PJK, 9CRV, 9D3C) and contain co-crystallized ligands within their binding sites (Figure S2a–e). The variation in binding site volumes (329.7–1545.4 Å^3^) and complexity of co-crystallized ligands reflects the structural diversity of neurotransmitter receptor families. BBB-permeant metabolites with diverse molecular sizes can be accommodated across this receptor panel that is represented by both ionotropic (GABRA2, GRIA2, GRIN2B) and metabotropic (DRD4, HTR1A) neurotransmitter receptor classes.

**Figure 6:**
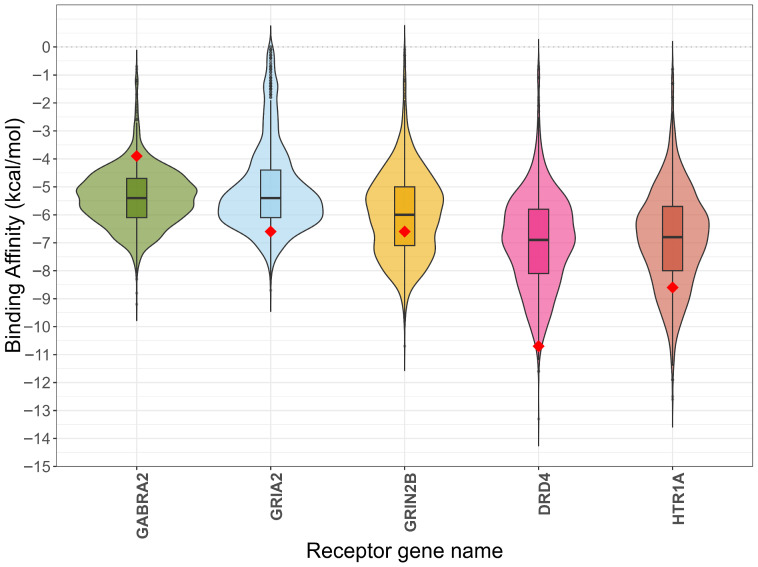
Comparative binding affinity distributions of BBB-permeant microbiome metabolites. Violin plots illustrate the density distribution of predicted binding affinities (kcal/mol) for 2239 BBB-permeant metabolites docked against the five neurotransmitter receptors. Each plot contains an embedded box plot (showing the median and quartiles) for statistical summary. The red diamonds indicate the binding affinity obtained from the re-docked co-crystallized ligand (positive control) for each receptor. BBB-permeant metabolites with positive binding scores were removed with the following counts: GABRA2 *n* = 2239, GRIA2 *n* = 1906, GRIN2B *n* = 2201, DRD4 *n* = 2216 and HTR1A *n* = 2239.

Molecular docking simulations were performed for 2244 BBB-permeant metabolites against the five selected neurotransmitter receptors, with 2239 ligands (99.8%) successfully completing the docking protocol (Figure 6). GABRA2 (PDB: 9CRV) and HTR1A (PDB: 8PJK) docked all screened metabolites with favorable (negative) binding affinities. In contrast, DRD4 (PDB: 5WIU), GRIN2B (PDB: 9D3C), and GRIA2 (PDB: 3RN8) had positive (repulsive) binding scores for subsets of metabolites, with 23, 38, and 333 metabolites showing repulsive interactions, respectively. These metabolites were removed for subsequent analysis. To validate the docking procedure, the co-crystallized ligand from each receptor structure was re-docked as a positive control (represented by red diamonds in Figure 6). The calculated binding affinities for these controls were highly reproducible: −6.6 kcal/mol for GRIA2, −10.7 kcal/mol for DRD4, −8.6 for HTR1A, −3.9 kcal/mol for GABRA2, and −6.6 kcal/mol for GRIN2B. Controls do not uniformly represent the strongest-binding ligands in each receptor. DRD4 control is only surpassed by 15 metabolites (0.7%) but GABRA2 control is surpassed by 2106 (94%). GRIA2, HTR1A and GRIN2B controls are surpassed by 184 (9.6%), 348 (15.5%) and 750 (34%) metabolites.

**Figure 7:**
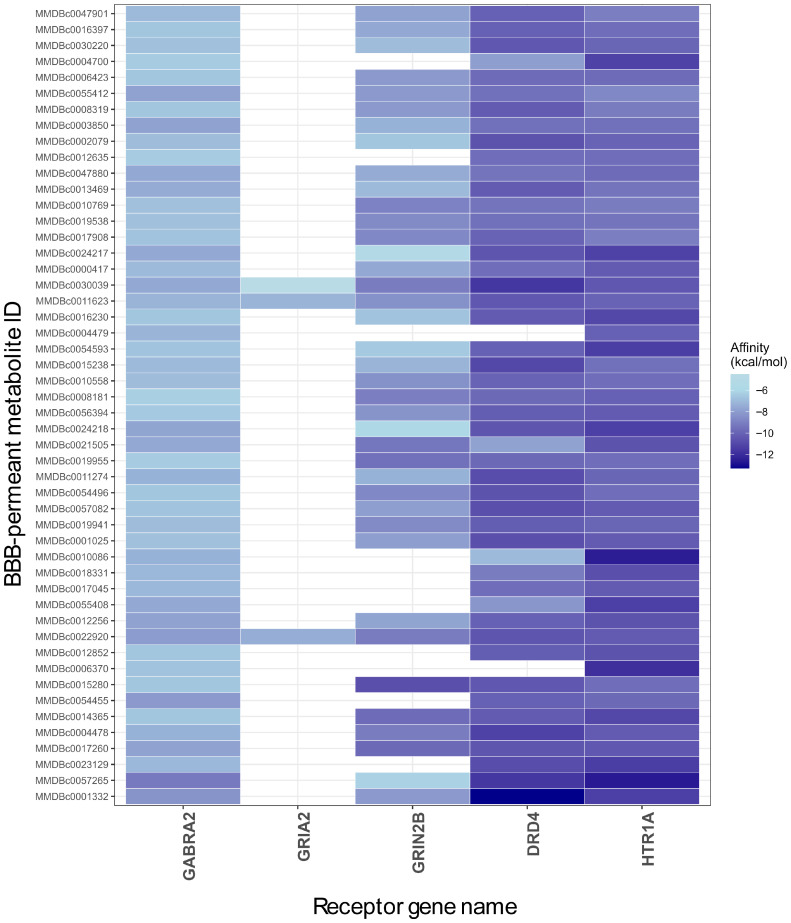
Heatmap of top 50 best-performing BBB-permeant metabolites across all receptors ranked by average affinity. Darker blue equals stronger affinities.

The top 50 best-performing BBB-permeant metabolites were identified by calculating the mean binding affinity for each metabolite across all five receptors (GABRA2, GRIA2, GRIN2B, DRD4, and HTR1A), ranking metabolites by these average values in ascending order and selecting the top 50 compounds with the lowest average affinities. These results are presented in the heatmap plot in Figure 7. Table 2 shows that their respective co-crystallized ligands of the ionotropic receptors GABRA2, GRIA2, and GRIN2B possess molecular volumes ranging from 96 to 220 Å^3^, whereas the metabotropic receptors DRD4 and HTR1A accommodate substantially larger ligands (341–369 Å^3^). Correspondingly, the binding site cavities of ionotropic receptors exhibit more constrained dimensions. This structural constraint is evident in the molecular docking results: GRIA2, possessing the smallest cavity volume (329.7 Å^3^), failed to accommodate 3 of the top 50 BBB-permeant metabolites identified for other receptors. GRIN2B demonstrated comparable selectivity, successfully docking 39 of the top 50 BBB-permeant metabolites. This improved accommodation can be attributed to its larger cavity volume (1054 Å^3^). Among the ionotropic receptors examined, GABRA2 was unique in its ability to dock all 50. Despite its cavity volume (450.8 Å^3^), the binding site of GABRA2 exhibits greater solvent accessibility (Figure S2d), thereby circumventing steric limitations imposed by larger ligand dimensions and permitting productive binding interactions without unfavorable steric clashes. Nevertheless, metabotropic receptors not only accommodated most of the top 50 BBB-permeant metabolites but also demonstrated superior binding affinities (<−10 kcal/mol). This comparative analysis of binding affinities across receptor subtypes suggests that ionotropic and metabotropic receptors should be evaluated as distinct classes in subsequent investigations.

From these top 50 BBB-permeant metabolites, 22 (44%) were classified by SwissADME, with 22 (44%) by PyTorch, and, importantly, only 6 (12%) were overlap metabolites identified by both methods. This limited concordance remarks the importance of employing multiple classification criteria and highlights the complementary nature of our predictive approaches for BBB permeability assessment.

**Figure 8:**
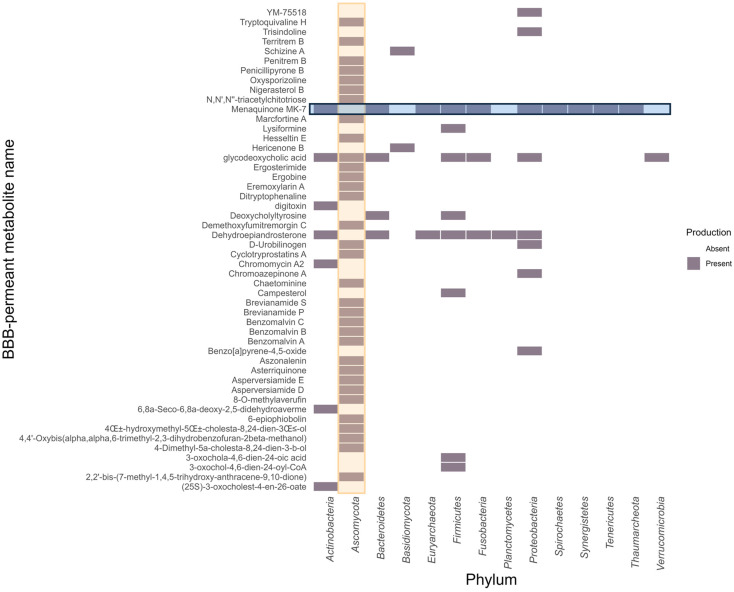
Phylum origins of top 50 BBB-permeant metabolites. Heatmap shows the phyla capable of producing each of the top 50 metabolites. *Ascomycota* (yellow highlight) produces 33 metabolites (66%), demonstrating fungal dominance. Menaquinone MK-7 (blue highlight) shows broad conservation across 10 phyla (71.4%).

To provide taxonomic context for the most active compounds, we analyzed the microbial origin of the top 50 BBB-permeant metabolites (those with highest predicted binding affinities across all receptors). The microbial origin by phylum of these top-ranking metabolites is presented in Figure 8. The *Ascomycota* phylum dominated this active set, contributing 33 of 50 metabolites (66%, highlighted in yellow in Figure 8), significantly higher than *Proteobacteria* and *Firmicutes* (8 metabolites each, 16%). This distribution reflects the underlying composition of the MiMeDB database: while *Ascomycota* organisms represent only 9.48% of cataloged microorganisms (251 of 2648, Table S2), they produce 62.3% of all metabolites in the database (18,245 out of 29,295 compounds in MiMeDB version 2, accessed November 2025). Our BBB-permeant compounds with high receptor binding affinity maintain this taxonomic prevalence, consistent with the underlying database composition rather than indicating differential BBB permeability across taxa. This observation highlights the relative underexploration of the gut mycobiome in neurodevelopmental research and emphasizes the need for taxonomically balanced metabolomic datasets to enable rigorous cross-taxa comparisons.

Also, menaquinone MK-7 (vitamin K2) metabolite, highlighted in blue in Figure 8, is present in 10 out of 14 (71.4%) phyla, followed by glycodeoxycholic acid and dehydroepiandrosterone, in 7 out of 14 (50%) phyla. This indicates metabolite evolutionary conservation through host–microbiome interaction. Both results have relevance for psychobiotic therapy that may confer mental health benefits by influencing the gut–brain axis in patients with TS, ADHD and ASD. Complete metabolite identifiers, common names, BBB-permeant classifiers and affinities for each receptor are provided in Table S1.

## 3. Discussion

The present study employed two fundamentally different computational strategies for BBB permeability prediction, yielding complementary metabolite sets with distinct physicochemical profiles. The SwissADME classification, based on the BOILED-Egg model, applies empirical rules derived from 660 experimentally characterized compounds using log*p* and TPSA as primary descriptors. This approach identified 1696 metabolites that conform to traditional medicinal chemistry constraints. The physicochemical profiles of these predicted BBB-permeant compounds—with a mean molecular weight of 243 ± 147 g/mol and a mean log*p* of 2.9 ± 2.2—fall well within Lipinski’s Ro5 boundaries. The conservative nature of this method reflects pharmaceutical industry experience, favoring drug-like molecules that exhibit restricted physicochemical properties for favorable ADME characteristics. However, a limitation of relying solely on this passive diffusion model is the potential for false negatives, as acknowledged by Daina and Zoete [57]. The log*p* and TPSA descriptors alone do not capture compounds that utilize active transport mechanisms or other non-passive diffusion pathways for BBB traversal. This inherent conservatism underscores the necessity of utilizing a complementary, data-driven model (e.g., our PyTorch network) to broaden the scope of virtual screening. Thus, the PyTorch neural network trained on 7807 experimental BBB permeability measurements identified 2484 metabolites spanning substantially broader chemical space. The model achieved 86.2% test accuracy with AUC 0.912, demonstrating robust discriminatory performance. PyTorch-classified metabolites exhibited mean molecular weight (929 ± 478 g/mol) and log*p* (12.9 ± 9.3) far exceeding traditional drug-likeness criteria. The dominance of TPSA found in feature importance analysis aligns with its established mechanistic role in BBB permeability, while the integrated contributions of secondary features support the “molecular complexity” factor interpretation derived from PCA. This PCA revealed a fundamental difference in how our two models operate. The PyTorch neural network training converged on a unidimensional “molecular complexity” factor that accounts for 92.2% of the total variance. This single learned representation efficiently integrates molecular size, lipophilicity, hydrogen bonding capacity, and aromaticity. This is in sharp contrast to the SwissADME model, which relies on a multi-dimensional descriptor space (PC1–3: 92.5% variance), reflecting its independent, rule-based filters. This divergence in approach is critical: SwissADME is a conservative method designed for drug development, restricting permeability predictions to compounds with favorable pharmacological indices. However, microbiome-derived metabolites are natural products and often violate Lipinski’s Ro5 restrictions. This algorithmic divergence is clearly evidenced by the limited overlap between the two classification methods: only 184 metabolites were positively classified by both (7.4% of the SwissADME total and 5.0% of the PyTorch total). This low consensus underscores the critical importance of employing multiple prediction approaches to maximize the therapeutic discovery space. The neural network’s capacity to identify larger, highly lipophilic metabolites as BBB-permeable suggests it captures alternative permeation mechanisms beyond simple passive transcellular diffusion. These mechanisms potentially include carrier-mediated transport or adsorptive-mediated transcytosis, which facilitate BBB crossing for compounds that violate Ro5. This complementarity is fully validated by analyzing the top 50 highest-affinity metabolites: 44% were identified exclusively by SwissADME, 44% exclusively by PyTorch, and only 12% by both methods. Relying on a single classification strategy would have failed to identify a significant portion of therapeutically promising candidates.

An important consideration in interpreting our docking results is that for GABRA2 and GRIN2B, 94% and 34% of screened metabolites, respectively, exhibited binding affinities more favorable than their native co-crystallized ligands. While this initially appears implausible, several factors explain this observation. First, the co-crystallized ligands (allosteric modulator for GABRA2, competitive antagonist for GRIN2B) are not necessarily optimized for maximal binding affinity; their therapeutic value derives from specific functional effects (partial agonism, selective antagonism) rather than pure binding strength. Methodological limitations of our study include that molecular docking provides binding affinity estimates but cannot establish functional consequences; metabolites may bind with high affinity yet lack efficacy or bind outside functional sites. The restriction to ligands with ≤32 rotatable bonds excluded 1715 metabolites. The receptor panel represents limited sampling; comprehensive assessment would require additional receptor subtypes, transporters, and metabolic enzymes. Selected structures represent single conformational states; alternative conformations may exhibit different binding profiles. Computational exploration of increased panels of neurotransmitter receptors and more conformational states with molecular dynamics simulations should be addressed to complement mechanistic insights of metabolites.

The phylogenetic distribution of top-performing BBB-permeant metabolites requires careful interpretation given the underlying database composition. The prominence of *Ascomycota*-derived compounds among high-affinity ligands (33 of 50 metabolites, 66%) directly mirrors their representation in the MiMeDB database (62.3% of all cataloged metabolites), indicating that this taxonomic distribution reflects annotation and sampling bias rather than differential BBB permeability or neuroactive compound production across microbial taxa. Consequently, these results should be interpreted as descriptive observations that cannot support comparative conclusions about the relative capacity of fungal versus bacterial metabolites to cross the BBB or modulate neurotransmitter receptors.

Nevertheless, the consistent identification of fungal-derived metabolites within the high-affinity subset, even if proportionally expected, highlights a notable gap in current microbiome–brain research, which has predominantly focused on bacterial contributions. The observed pattern generates testable hypotheses for future investigation. For instance, elevated *Ascomycota* abundance documented in some ASD cohorts [58,59] could reflect either dysbiotic states or compensatory responses. Distinguishing between these scenarios requires species-level characterization of *Ascomycota* communities in ASD patients combined with strain-specific metabolite profiling to identify which fungal species produce therapeutically relevant metabolites. The representation of *Firmicutes*-derived metabolites (8 of 50, 16%) relates to alterations in *Firmicutes* abundance and *Firmicutes/Bacteroidetes* ratios that have been consistently documented in ASD cohorts, though the direction of change varies across studies [18,20]. If specific *Firmicutes* strains producing these high-affinity metabolites are altered in ASD patients, changes in metabolite availability at neurotransmitter receptors could represent a mechanistic link between documented microbial dysbiosis and neurobiological dysfunction. This hypothesis is testable through metabolomic profiling comparing plasma concentrations of identified *Firmicutes*-derived metabolites between ASD patients and neurotypical controls, with alterations potentially addressable through targeted probiotic supplementation with specific metabolite-producing strains.

The broad conservation of menaquinone MK-7 production across 10 of 14 phyla (71.4%) suggests evolutionary selection for production of this metabolite, potentially reflecting fundamental importance in host–microbiome interactions. Notably, vitamin K2 (menaquinone) plays crucial roles in the central nervous system beyond vitamin K-dependent carboxylation reactions, including preservation of tyrosine hydroxylase expression—the rate-limiting enzyme for dopamine synthesis—and activation of neuroprotective proteins Gas6 and protein S [60,61]. Given that dopaminergic dysfunction is implicated across TS, ADHD, and ASD, and that children with ASD exhibit lower serum menaquinone-4 concentrations compared to neurotypical controls, the identification of MK-7 as a high-affinity ligand produced by diverse microbial phyla warrants investigation of its potential therapeutic role across NDDs [16,62,63].

The identification of microbiome metabolites with high binding affinity to DRD4 (median −6.9 kcal/mol, the strongest among evaluated receptors) and GABRA2 provides targets for addressing both dopaminergic excess and GABAergic-mediated motor disinhibition underlying pathophysiology. The predominance of multi-receptor binding metabolites offers additional therapeutic value for TS, where 50–60% of patients exhibit comorbid ADHD and 20–30% show ASD features [64,65,66], enabling simultaneous modulation of dopaminergic, GABAergic, and serotonergic systems relevant to tics, compulsive behaviors, and comorbid symptoms through single-strain or multi-strain probiotic formulations.

MiMeDB aggregates metabolites from across the human-associated microbiome, including many compounds detected exclusively in stool samples without confirmed systemic circulation. While some of these metabolites may reach host tissues via intestinal absorption, vagus-nerve-mediated signaling, or microbial translocation, others may not enter the bloodstream. Therefore, BBB-permeability predictions for metabolites lacking evidence of circulation should be interpreted with caution. This limitation is inherent to applying computational permeability models to whole-microbiome databases and is now explicitly noted in this study.

The metabolite–receptor interaction results establish a basis for precision medicine approaches where interventions are tailored to individual patients’ neurobiological profiles. Current probiotic interventions employ generic formulations without consideration of individual metabolomic status or specific neurotransmitter dysfunctions, likely contributing to inconsistent clinical trial results and high inter-individual variability in treatment response. Metabolomic profiling of plasma, urine, or fecal samples from individual patients could identify specific metabolite deficiencies or excesses relative to neurotypical controls. For instance, patients exhibiting deficits in dopamine receptor ligands might benefit from strains producing metabolites with DRD4 binding affinity, while those showing glutamate/GABA imbalance could receive strains producing GABRA2 or GRIN2B modulators. The identification of metabolites exhibiting multi-receptor binding capacity offers therapeutic value given substantial comorbidity across TS, ADHD, and ASD, potentially addressing diverse symptom domains through coordinated neurotransmitter modulation [28,29].

These findings open a translational pathway for future investigations including experimental validation of BBB permeability through in vitro transwell models and in vivo pharmacokinetics; functional receptor assays to determine agonist, antagonist, or allosteric modulator activity; identification of metabolite-producing gut strains at therapeutically relevant concentrations; behavioral validation in NDD animal models comparing native versus metabolite-producing strain colonization [22,23]; and developmental neurotoxicity assessment and pilot clinical trials using metabolomic profiling to stratify patients by baseline metabolite deficiencies and correlate metabolite changes with symptom improvement.

## 4. Materials and Methods

### 4.1. Microbiome Dataset and Blood–Brain Barrier Permeability Classification

The complete microbiome metabolite database MiMeDB (www.mimedb.org), including 27,642 microbiome (accessed in September 2025) SMILES metabolites, was downloaded and processed using SMILES (Simplified Molecular Input Line Entry System) format for subsequent cheminformatics analysis. MiMeDB is a comprehensive microbiome metabolite database that compiles small molecules produced or associated with human-associated microorganisms. Importantly, MiMeDB includes both metabolites detected in human samples and compounds whose biological occurrence or circulation status has not yet been experimentally confirmed. We employed the complete MiMeDB database to capture the full spectrum of microbial metabolites, including those not yet characterized in systemic circulation. Two complementary approaches were implemented to filter MiMeDB for blood–brain barrier (BBB) permeability: manual curation using the SwissADME [67] web-based platform (www.swissadme.ch), and machine learning classification using a neural network implemented in PyTorch.

For manual curation in SwissADME, SMILES codes were uploaded to a web platform; the BOILED-Egg model’s default BBB permeability prediction was utilized, which is based on compounds falling within the ‘yolk’ region defined by log*p* and TPSA values. For the neural network approach, a curated experimental BBB dataset (BBB_DB) comprising 7807 compounds labeled as BBB-permeant or non-permeant was employed for model training [68]. Molecular descriptors were computed using the RDKit library [69], including molecular weight, log*p*, total polar surface area (TPSA), atom count, hydrogen bond donors (HBD), hydrogen bond acceptors (HBA), number of rotatable bonds, aromatic atom count, and ring count. These descriptors served as input features for both BBB_DB training and subsequent MiMeDB classification.

The neural network architecture consisted of a feedforward multilayer perceptron with three hidden layers (20, 15, and 4 neurons) and sigmoid activation functions. A dropout rate of 0.01 was implemented for regularization. The model was trained using the Adam optimizer with an initial learning rate of 0.01 and binary cross-entropy loss function. Training employed a reducing learning rate (ReduceLROnPlateau) scheduler (factor = 0.5, patience = 5 epochs) and early stopping (patience = 20 epochs) based on validation loss. The BBB_DB dataset (*n* = 7807) was split into training (70%, *n* = 5465), validation (10%, *n* = 781), and test (20%, *n* = 1561) sets using stratified sampling to maintain BBB-permeant and BBB-non-permeant class balance. Min–max normalization was applied to all features, and missing values were imputed using mean imputation. Training was performed with a batch size of 32 over a maximum of 1000 epochs. Feature importance was evaluated using permutation-based analysis, where each molecular descriptor was randomly shuffled in the test set (10 permutations per feature) and the resulting decrease in prediction accuracy was measured. The importance score represents the mean reduction in model accuracy when a feature’s information is disrupted, with higher values indicating stronger predictive contributions. All computations were conducted on an Ubuntu 24.04 LTS workstation (Santa Clara, CA, USA) equipped with an NVIDIA RTX 5070 GPU for hardware acceleration.

Because rule-based filtering can constrain the chemical space in ways that depend on user-defined parameters, we paired SwissADME with an independent, data-driven classifier (hereafter referred to as SwissADME and PyTorch methods). The PyTorch approach expands the assessment from the two permeability-related rules used by SwissADME to nine molecular descriptors, allowing a broader and more nuanced characterization of BBB permeability-related chemical features. Using orthogonal methods therefore mitigates the risk of excluding metabolites simply because they do not match a particular rule set. This expands the explored chemical space and provides convergent evidence when both approaches agree. Therefore, chemical descriptors of BBB-permeant metabolites identified from MiMeDB obtained by both filtering approaches were analyzed by Lipinski Ro5, compared with density plots and principal component analysis using the R programming language.

Claude Sonnet 4.5 (Anthropic, San Francisco, CA, USA, 2025) was used to provide guidance on Python 3.12.3 and R 4.3.1 pipeline development, including workflow architecture and package selection. All generated code and recommendations were independently reviewed, tested, and validated by the authors and are provided in the Supplementary Information at FigShare (https://doi.org/10.6084/m9.figshare.30494093).

### 4.2. Molecular Screening

To identify microbiome metabolites capable of interacting with neurotransmitter receptors implicated in Tourette syndrome (TS), attention-deficit/hyperactivity disorder (ADHD), and autism spectrum disorder (ASD), a docking screening approach was implemented. Neurotransmitter receptors associated with these neuropsychiatric disorders were identified through the UniProt database (www.uniprot.org) using the search term “neurotransmitter receptor” with filters applied for three-dimensional (3D) structure availability and annotated binding sites. Subsequent manual curation was performed by cross-referencing the Protein Data Bank (PDB; www.rcsb.org) to ensure only experimentally determined structures were included. When multiple PDB entries were available for a given receptor, structures were selected based on the following hierarchical criteria: (i) receptor implication in more than one neurodevelopmental disorder, (ii) maximum coverage of the canonical protein sequence, (iii) presence of a co-crystallized ligand within the binding site, and (iv) optimal PDB validation metrics.

Protein structures were prepared by removing non-binding-site ligands, metal ions, and solvent molecules. Explicit hydrogen atoms were added using UCSF ChimeraX [56], and missing internal loops were modeled using Modeller 10.8 [70] package. BBB-crossing metabolites identified from MiMeDB were subjected to 3D structure optimization using the Universal Force Field (UFF) with explicit hydrogens via the OpenBabel program.

Molecular docking simulations were performed using AutoDock Vina [33]. Ligand and receptor structures were converted to PDBQT format with Gasteiger partial charges assigned. The docking grid box was centered on the coordinates of the co-crystallized ligand for each receptor structure. Default AutoDock Vina parameters were employed for molecular screening, and the top-scoring binding pose was selected for subsequent analysis.

## 5. Conclusions

This computational study successfully established a comprehensive virtual screening pipeline to identify high-affinity BBB-permeant metabolites from the MiMeDB capable of binding a panel of five neurotransmitter receptors implicated in TS, ADHD and ASD. The final selection process, based on the intersection of two distinct BBB classification approaches (SwissADME and PyTorch neural network), validated the robustness of our candidate set. The comparison between the two BBB-permeant classifiers revealed crucial methodological insights: the SwissADME model adheres to strict, rule-based physicochemical constraints, while the PyTorch neural network demonstrated a capacity to classify compounds with a broader range of physicochemical properties. This complementarity is essential, as evidenced by the equal distribution of top-binding results across both classifiers. The identification of metabolites exhibiting multi-receptor binding profiles provides promising candidates for broad-spectrum symptom management. Furthermore, the dominance of the *Ascomycota* phylum among the top metabolites (66%) underscores the urgent need to expand research focus to the gut mycobiome and its role in NDDs. Collectively, these findings establish detailed receptor–metabolite interaction maps and prioritize candidates for immediate experimental validation and targeted strain selection. Furthermore, the predominance of *Ascomycota*-derived metabolites among the top-ranking compounds (66%) reflects the current composition and annotation bias of the MiMeDB database rather than an inferred biological advantage in BBB permeability. Accordingly, these results should be interpreted as descriptive and hypothesis-generating. Nevertheless, the consistent presence of fungal-derived metabolites within the high-affinity BBB-permeant subset highlights the relative underexploration of the gut mycobiome in neurodevelopmental research and underscores the need for more balanced, taxonomically comprehensive metabolomic datasets. Future studies incorporating normalized, species-level metabolite coverage will be essential to rigorously assess comparative BBB permeability across microbial taxa. Taken together, this in silico study provides a hypothesis-generating framework for precision medicine approaches, suggesting that future patient-specific metabolomic profiling could inform the development of personalized, microbiome-targeted therapeutics. This perspective opens a novel avenue to address significant clinical needs in NDDs, subject to experimental validation.

## Figures and Tables

**Table 1:** Statistical analysis (mean ± SD) of nine molecular descriptors for metabolites classified as BBB-permeant by SwissADME and PyTorch. Data summarizes the distribution of physicochemical properties used in the comparative density analysis shown in Figure 3.

Chemical Descriptor	SwissADME Mean ± SD	PyTorch Mean ± SD
Molecular weight (g/mol)	243.1 ± 146.5	928.7 ± 478
log*p*	2.9 ± 2.2	12.9 ± 9.3
TPSA (Å^2^)	48.2 ± 42.2	184.2 ± 92.5
Atom count	17 ± 10	63 ± 33
HBD	1 ± 1	3 ± 2
HBA	3 ± 2	11 ± 6
Rotatable bonds	5 ± 6	45 ± 30
Aromatic atoms	1 ± 1	0 ± 1
Number of rings	2 ± 1	1 ± 1

**Table 2:** Selected PDBs for molecular screening.

Gene Name	PDB ID	Crystalized Domain	Canonical Sequence Length	PDB Sequence Length	Binding Site Volume (Å^3^) *	Co-Crystalized Ligand Volume (Å^3^) **	Reference
GABRA2	9CRV	All	451	451	450.8	96	[50]
GRIA2	3RN8	Ligand binding	883	280	329.7	125.5	[51]
GRIN2B	9D3C	Ligand binding	1484	884	1054.4	220.1	[52]
DRD4	5WIU	All	419	419	1274.4	369.8	[53]
HTR1A	8PJK	Ligand binding	422	352	1545.4	341.9	[54]

* Volumes calculated with Fpocket program 4.0 [55]. ** Volumes calculated with UCSF ChimeraX 1.19 [56].

## Data Availability

All generated code and data is provided at FigShare (https://doi.org/10.6084/m9.figshare.30494093).

## Supplementary Materials

The following supporting information can be downloaded at https://www.mdpi.com/article/10.3390/molecules31020366/s1: Figure S1: SwissADME (pink bars) versus PyTorch (blue bars) preferences for BBB-crossing metabolites classification according to Lipinski’s Rule of Five (Ro5). Frequency overlap in bars is represented in transparency; Figure S2. Crystallized ligands in their binding site for (a) GRIA2 (PDB: 3RN8), (b) DRD4 (PDB: 5WIU), (c) HTR1A (PDB: 8PJK), (d) GABRA2 (PDB: 9CRV) and (e) GRIN2B (PDB: 9D3C); Figure S3. Permutation-based feature importance analysis for the PyTorch BBB permeability classifier. Bar plot showing the mean decrease in model accuracy when each molecular descriptor was randomly permuted in the test set (*n* = 10 permutations per feature). Error bars represent standard deviation across permutations. Table S1: MiMeDB IDs from top 50 BBB-permeant metabolites and its common molecule name, the phylum of production origin, the BBB-permeant classifier that filtered them and their binding affinity across all 5 receptors evaluated by molecular docking; Table S2. Distribution of Phylum in MiMeDB.

## Abbreviations

The following abbreviations are used in this manuscript:

BBB	Blood–Brain Barrier
ROC	Receiver Operating Characteristic
MiMeDB	Microbiome Metabolite Database
TPSA	Total Polar Surface Area
HBD	Hydrogen Bond Donnor
HBA	Hydrogen Bond Acceptor
Ro5	Lipinski Rule of Five
PCA	Principal Component Analysis
ADHD	Attention-Deficit Hyperactivity Disorder
ASD	Autism Spectrum Disorder
GABRA2	Gamma-aminobutyric Acid Receptor subunit Alpha 2
DRD4	Dopamine Receptor 4
HTR1A	Serotonin Receptor 1A
AMPA	Alpha-amino-3-hydroxy-5-methyl-4-isoxazolepropionic Acid
GRIA2	AMPA receptor 2
NMDA2/GRIN2B	*N*-methyl-D-aspartate receptor subunit 2B
UFF	Universal Force Field
