# Supplementary material for: Computational Identification of Blood–Brain Barrier-Permeant Microbiome Metabolites with Binding Affinity to Neurotransmitter Receptors in Neurodevelopmental Disorders
Source: Molecules. 2026 Jan 20;31(2):366. doi: 10.3390/molecules31020366 (PMC12844128; doi:10.3390/molecules31020366)
Supplement: Supplementary file 1 [file molecules-31-00366-s001.zip › molecules-4004418-supplementary.pdf]

Supplementary information

Computational Identification of Blood-Brain Barrier permeant Microbiome Metabolites with Binding Affinity to Neurotransmitter Receptors in Neurodevelopmental Disorders

Ricardo E. Buendia-Corona[1], María Fernanda Velasco Dey[2], Lisset Valencia Robles[2], Hannia Josselín Hernández-Biviano[3], Cristina Hermosillo-Abundis[2], Lucila Isabel Castro-Pastrana[4*]

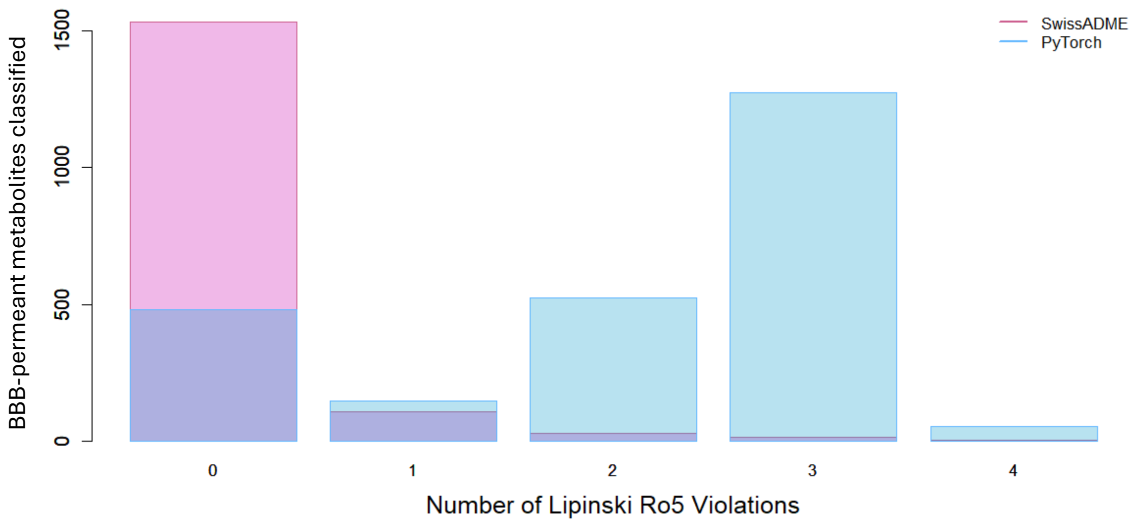

Figure S1. SwissADME (pink bars) versus PyTorch (blue bars) preferences for BBB-crossing metabolites classification according to Lipinski's Rule of Five (Ro5). Frequency overlap in bars is represented in transparency.

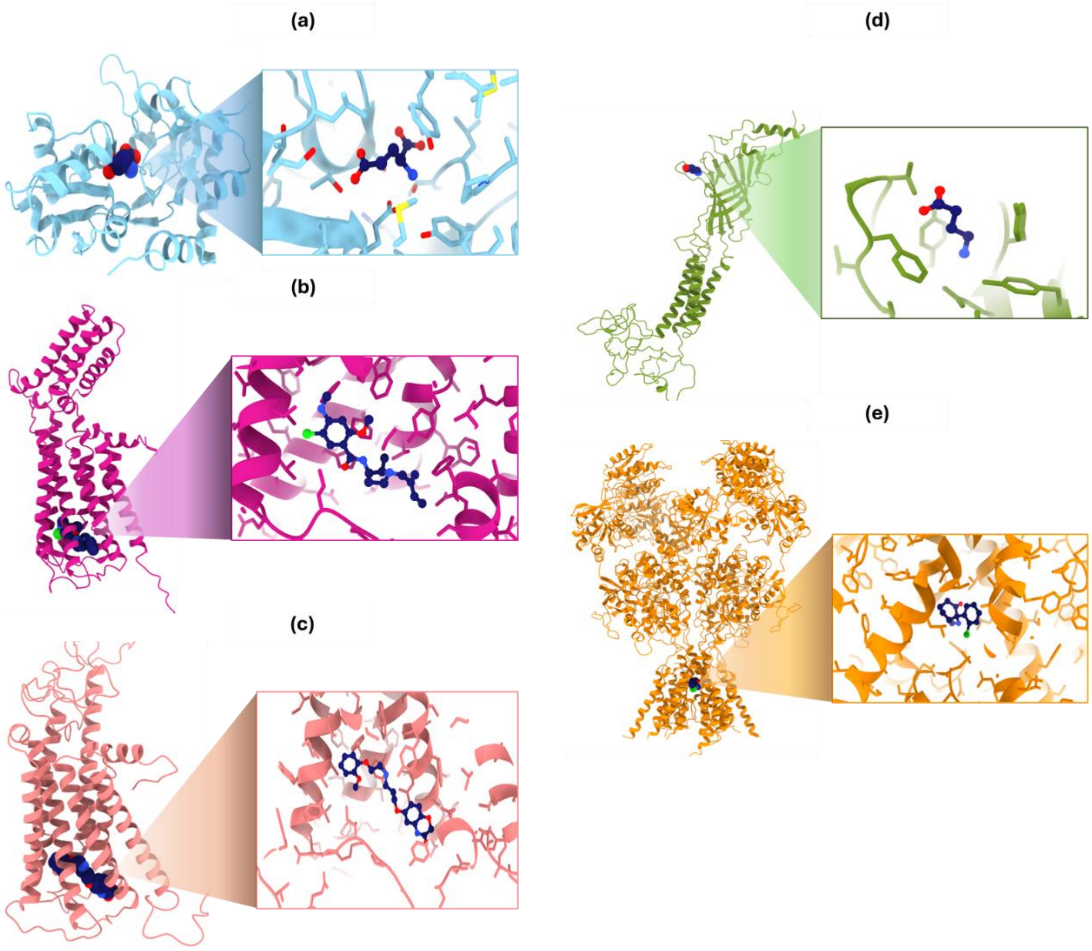

Figure S2. Crystallized ligands in their binding site for a) GRIA2 (PDB: 3RN8), b) DRD4 (PDB: 5WIU), c) HTR1A (PDB: 8PJK), d) GABRA2 (PDB: 9CRV) and e) GRIN2B (PDB: 9D3C).

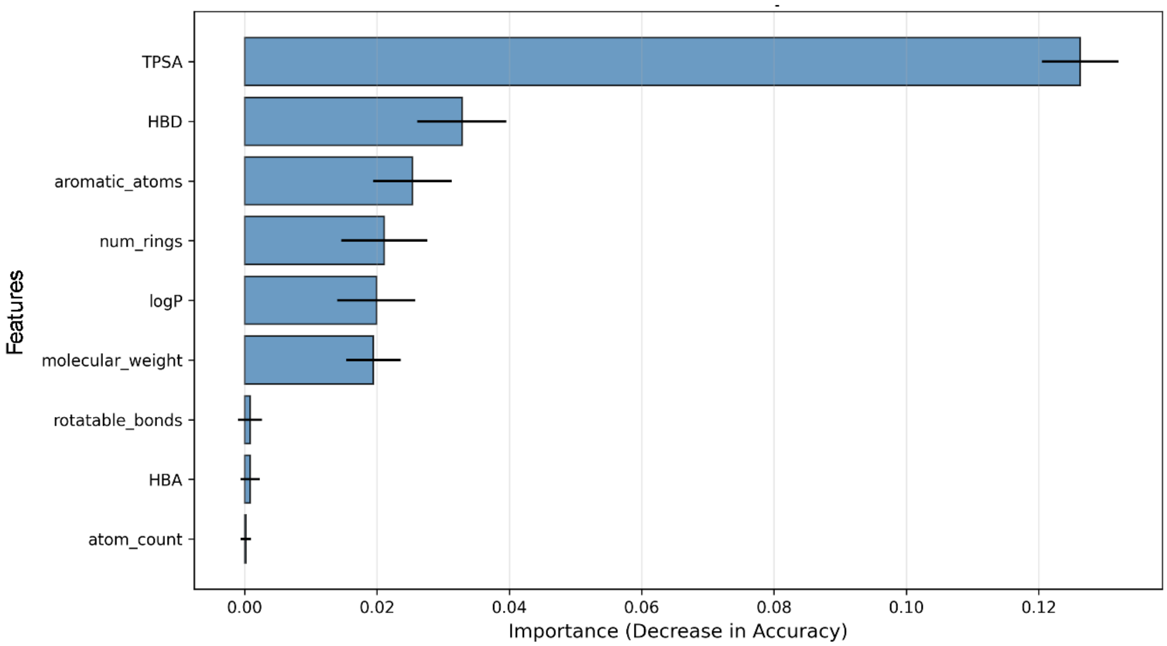

Figure S3. Permutation-based feature importance analysis for the PyTorch BBB permeability classifier. Bar plot showing the mean decrease in model accuracy when each molecular descriptor was randomly permuted in the test set (n = 10 permutations per feature). Error bars represent standard deviation across permutations.

Table S1. MiMeDB IDs from top 50 BBB-permeant metabolites and its common molecule name, the phylum of production origin, the BBB-permeant classifier that filtered them and their binding affinity across all 5 receptors evaluated by molecular docking.

ID	Name	*Phylum*	BBB Classifier	Affinity in kcal/mol				
				GABRA2	GRIA2	GRIN2B	DRD4	HTR1A
**MMDBc0047901**	Dehydroepiandrosterone	*Proteobacteria, Bacteroidetes, Firmicutes, Actinobacteria, Fusobacteria, Euryarchaeota, Planctomycetes*	Overlap	-7	1.7	-7.8	-10.1	-9
**MMDBc0016397**	Hesseltin E	*Ascomycota*	Overlap	-6.6	8.6	-7.6	-10.1	-9.6
**MMDBc0030220**	D-Urobilinogen	*Proteobacteria, Ascomycota*	PyTorch	-6.8	0.8	-6.9	-10.4	-9.9
**MMDBc0004700**	Penitrem B	*Ascomycota*	PyTorch	-6.4	32.6	9.2	-7.9	-11.2
**MMDBc0006423**	6-epiophiobolin	*Ascomycota*	SwissADME	-6.6	11.3	-8.1	-9.7	-9.7
**MMDBc0055412**	3-oxochola-4,6-dien-24-oic acid	*Firmicutes*	SwissADME	-7.8	2.5	-8.1	-9.5	-8.7
**MMDBc0008319**	Penicillipyrone B	*Ascomycota*	SwissADME	-6.6	2.2	-8.1	-10.3	-9.1
**MMDBc0003850**	4,4'-Oxybis(alpha,alpha,6-trimethyl-2,3-dihydrobenzofuran-2beta-methanol)	*Ascomycota*	SwissADME	-7.8	0.1	-7.3	-9.5	-9.5
**MMDBc0002079**	8-O-methylaverufin	*Ascomycota*	PyTorch	-6.9	1.1	-6.6	-10.6	-10
**MMDBc0012635**	Territrem B	*Ascomycota*	PyTorch	-6.4	15.1	2.7	-9.6	-9.6
**MMDBc0047880**	4Œ±-hydroxymethyl-5Œ±-cholesta-8,24-dien-3Œ≤-ol	*Ascomycota*	PyTorch	-7.6	2.9	-7.5	-9.4	-9.7
**MMDBc0013469**	Cyclotryprostatins A	*Ascomycota*	PyTorch	-7.5	11.8	-7	-10.3	-9.4
**MMDBc0010769**	Nigerasterol B	*Ascomycota*	SwissADME	-6.8	12.1	-8.9	-9.4	-9.1
**MMDBc0019538**	Eremoxylarin A	*Ascomycota*	PyTorch	-6.8	7.4	-8.6	-9.5	-9.4
**MMDBc0017908**	Aszonalenin	*Ascomycota*	SwissADME	-6.7	9.9	-8.7	-10	-9

**MMDBc0024217**	Asperversiamide D	*Ascomycota*	SwissADME	-7.6	21.4	-5.2	-10.5	-11.2
**MMDBc0000417**	Campesterol	*Firmicutes*	PyTorch	-7	3.1	-7.6	-9.6	-10.3
**MMDBc0030039**	Benzo[a]pyrene-4,5-oxide	*Proteobacteria*	Overlap	-7.6	-4.5	-9.1	-11.6	-10.4
**MMDBc0011623**	Hericenone B	*Basidiomycota*	SwissADME	-7.2	-7.2	-8.4	-10.4	-10
**MMDBc0004479**	6,8a-Seco-6,8a-deoxy-2,5-didehydroaverme	*Actinobacteria*	SwissADME	-7.2	108.4	27	7.3	-10.1
**MMDBc0016230**	Ergosterimide	*Ascomycota*	PyTorch	-6.6	8.1	-6.7	-10.3	-11
**MMDBc0054593**	N,N',N''-triacetylchitotriose	*Ascomycota*	PyTorch	-6.7	10.7	-6.5	-10.1	-11.4
**MMDBc0015238**	Demethoxyfumitremorgin C	*Ascomycota*	Overlap	-7	1.9	-7.2	-11	-9.5
**MMDBc0010558**	Brevianamide P	*Ascomycota*	SwissADME	-6.9	2.3	-8.4	-10	-9.6
**MMDBc0008181**	Schizine A	*Basidiomycota*	SwissADME	-6.3	27.5	-9	-9.8	-10.1
**MMDBc0056394**	(25S)-3-oxocholest-4-en-26-oate	*Actinobacteria*	SwissADME	-6.5	0.6	-8.3	-10.2	-10.3
**MMDBc0024218**	Asperversiamide E	*Ascomycota*	SwissADME	-7.7	13.8	-5.8	-10.5	-11.3
**MMDBc0021505**	Oxysporizoline	*Ascomycota*	SwissADME	-7.6	18.2	-9.3	-7.8	-10.6
**MMDBc0019955**	Chromoazepinone A	*Proteobacteria*	PyTorch	-6.4	2.1	-9.5	-9.8	-9.6
**MMDBc0011274**	YM-75518	*Proteobacteria*	Overlap	-7.3	16.4	-7.3	-10.8	-9.9
**MMDBc0054496**	glycodeoxycholic acid	*Actinobacteria, Firmicutes, Ascomycota, Proteobacteria, Bacteroidetes, Fusobacteria, Verrucomicrobia*	PyTorch	-6.6	1.4	-8.7	-10.6	-9.6
**MMDBc0057082**	4-Dimethyl-5a-cholesta-8,24-dien-3-b-ol	*Ascomycota*	PyTorch	-6.7	3.2	-7.9	-10.7	-10.3
**MMDBc0019941**	Benzomalvin B	*Ascomycota*	SwissADME	-6.9	18.2	-8.6	-10.2	-9.9

**MMDBc0001025**	Menaquinone MK-7	*Firmicutes, Proteobacteria, Actinobacteria, Bacteroidetes, Spirochaetes, Thaumarcheota, Fusobacteria, Synergistetes, Euryarchaeota, Tenericutes*	Overlap	-6.8	8.3	-7.9	-10.7	-10.3
**MMDBc0010086**	Ditryptophenaline	*Ascomycota*	PyTorch	-7.3	41.2	3.7	-7	-12.5
**MMDBc0018331**	Marcfortine A	*Ascomycota*	SwissADME	-7.1	24.8	5.8	-9.1	-10.7
**MMDBc0017045**	Brevianamide S	*Ascomycota*	SwissADME	-7.1	16.1	6	-9.6	-10.3
**MMDBc0055408**	3-oxochol-4,6-dien-24-oyl-CoA	*Firmicutes*	PyTorch	-7.6	40.6	7.5	-8.2	-11.3
**MMDBc0012256**	Benzomalvin C	*Ascomycota*	SwissADME	-7.8	8.4	-7.7	-10.1	-10.6
**MMDBc0022920**	Lysiformine	*Firmicutes*	SwissADME	-8	-7.4	-9.1	-10.5	-10.3
**MMDBc0012852**	Asterriquinone	*Ascomycota*	PyTorch	-6.6	9.1	0.3	-10.2	-10.6
**MMDBc0006370**	Chromomycin A2	*Actinobacteria*	PyTorch	-6.7	65.8	15.7	4.4	-11.9
**MMDBc0015280**	Chaetominine	*Ascomycota*	PyTorch	-6.6	2.9	-10.7	-10.4	-9.6
**MMDBc0054455**	Digitoxin	*Actinobacteria*	SwissADME	-8.1	27.5	0.8	-10.1	-9.8
**MMDBc0014365**	Tryptoquivaline H	*Ascomycota*	PyTorch	-6.6	6.8	-9.7	-10.3	-11
**MMDBc0004478**	Benzomalvin A	*Ascomycota*	SwissADME	-7.3	6.8	-9.1	-11.2	-10.3
**MMDBc0017260**	Trisindoline	*Proteobacteria*	SwissADME	-7.8	1.5	-9.8	-10.5	-10.4
**MMDBc0023129**	2,2'-bis-(7-methyl-1,4,5-trihydroxy-anthracene-9,10-dione)	*Ascomycota*	PyTorch	-7.1	13	6.7	-10.8	-11.4
**MMDBc0057265**	Deoxycholyltyrosine	*Bacteroidetes, Firmicutes*	PyTorch	-9.2	2.6	-6.2	-11.6	-12.6
**MMDBc0001332**	Ergobine	*Ascomycota*	PyTorch	-8.3	11.3	-8.1	-13.3	-11.3

Table S2. Distribution of Phylum in MiMeDB.

Phylum	n	Percentage
Firmicutes	849	32.06
Proteobacteria	583	22.02
Actinobacteria	399	15.07
Ascomycota	251	9.48
Bacteroidetes	219	8.27
Basidiomycota	63	2.38
Euryarchaeota	46	1.74
Fusobacteria	36	1.36
Tenericutes	31	1.17
Bacillota	29	1.1
NULL	15	0.57
Spirochaetes	14	0.53
Uroviricota	13	0.49
Crenarchaeota	9	0.34
Cyanobacteria	8	0.3
Chlamydiae	7	0.26
Mucoromycota	6	0.23
Nematoda	6	0.23
Platyhelminthes	6	0.23
Synergistetes	6	0.23
Chlorobi	4	0.15
Peploviricota	4	0.15
Bacteroidota	3	0.11
Verrucomicrobia	3	0.11
Actinomycetota	2	0.08
Apicomplexa	2	0.08
Candidatus thermoplasmatota	2	0.08
Chloroflexi	2	0.08
Chlorophyta	2	0.08
Chytridiomycota	2	0.08
Deferribacteres	2	0.08
Deinococcus-thermus	2	0.08
Ignavibacteriae	2	0.08
Microsporidia	2	0.08
Planctomycetes	2	0.08
Thermotogae	2	0.08
Aquificae	1	0.04
Candidatus melainabacteria	1	0.04

Ciliophora	1	0.04
Cossaviricota	1	0.04
Cressdnaviricota	1	0.04
Euglenozoa	1	0.04
Fibrobacteres	1	0.04
Hofneiviricota	1	0.04
Negarnaviricota	1	0.04
Nitrososphaerota	1	0.04
Nitrospirae	1	0.04
Oomycota	1	0.04
Pseudomonadata	1	0.04
Thaumarchaeota	1	0.04
